# A Cascade of Champions: A Qualitative Study about the MA-CORD Media Competition Implementation

**DOI:** 10.3390/ijerph13040404

**Published:** 2016-04-05

**Authors:** Shaniece Criss, Alvin Tran, Claudia Ganter, Alyssa Aftosmes-Tobio, Steven Gortmaker, Kasisomayajula Viswanath, Jo-Ann Kwass, Kirsten K. Davison

**Affiliations:** 1 Department of Social and Behavioral Sciences, Harvard T.H. Chan School of Public Health, 677 Huntington Avenue, Kresge Building, Boston, MA 02115, USA; sgortmak@hsph.harvard.edu (S.G.); vish_viswanath@dfci.harvard.edu (K.V.); kdavison@hsph.harvard.edu (K.K.D.); 2 Department of Nutrition, Harvard T.H. Chan School of Public Health, 655 Huntington Avenue, Boston, MA 02115, USA; alvintran@mail.harvard.edu (A.T.); cgehre@hsph.harvard.edu (C.G.); aaftosme@hsph.harvard.edu (A.A.-T.); 3 Dana-Farber Cancer Institute, 450 Brookline Avenue, Boston, MA 02215, USA; 4 Massachusetts Department of Public Health, Bureau of Community Health and Prevention, 250 Washington Street, Boston, MA 02108, USA; jo-ann.kwass@state.ma.us

**Keywords:** media competition, elementary school, implementation, qualitative, ecological framework for understanding effective implementation

## Abstract

A media competition was part of the Massachusetts Childhood Obesity Research Demonstration (MA-CORD) Study. Criss *et al.*, previously outlined the development and implementation of the competition, including variation in reach and adoption of the intervention across schools and afterschool programs. In this qualitative study, we examine community, provider, and organizational factors that explain the variation of media competition reach in school and afterschool programs, and describe the awareness of the media competition across other community sectors. Durlak and DuPre’s ecological framework for understanding effective implementation provided the theoretical underpinnings for this study. Fifty-four key informant interviews were conducted, transcribed, and analyzed. Organizational capacity of committed teachers/staff and adaptability of the media competition seemed to be drivers for higher reach within school and afterschool programs. Salient themes that emerged as facilitators of effective implementation were having a cascade of champions and providing opportunity to participate in the media competition outside traditional class time. Clinics and coalitions were identified as additional sectors aware of the media competition. Specifically, our findings offer a new perspective on intervention design and a recommended direction for further study.

## 1. Introduction

A great deal of effort is being directed toward childhood obesity interventions, and it is paramount that researchers understand how their interventions will translate into real-world settings. To increase the likelihood of success, it is crucial that such interventions are implemented effectively; a critical aspect is that they reach the intended audience [1]. The implementation process can influence outcomes in childhood obesity prevention in schools [2]. Therefore, researchers need to conduct process evaluations to disentangle whether a program’s success or failure was due to intervention design or implementation [3]. Understanding the extent to which programs are implemented effectively and drivers of effective implementation, including facilitators and barriers, can support the translation of studies into application.

The Massachusetts Childhood Obesity Research Demonstration Study (MA-CORD), known as *Mass in Motion Kids*, was a multi-level, multi-sector community intervention to prevent and reduce childhood obesity among low-income children, aged 2–12 years in two communities in Massachusetts (MA) from August 2012 to August 2014 [4,5], and it was funded by the Centers for Disease Control and Prevention as part of a comprehensive approach in several cities across the U.S. to address childhood obesity [6]. Sectors engaged in MA-CORD included healthcare, early care and education, the Supplemental Nutrition Program for Women, Infants, and Children program (WIC), schools, afterschool programs, and the broader community. This study focuses on the implementation of the media competition component of MA-CORD in 18 school and afterschool programs, which had 595 student participants. Elementary and middle school aged students developed videos, song/rap lyrics, and artwork that reflected “How can you be a Mass in Motion Kid?” by addressing MA-CORD goals concerning nutrition, physical activity, screen time, and sleep. Criss *et al.*, described the process used to develop and facilitate the media competition along with the results of implementation measures of adoption and reach [7]. That study found that there was tremendous variation in reach of the media competition: seven schools and afterschool programs demonstrated low reach, nine demonstrated moderate reach, and two demonstrated high reach (level of reach will be explained in the methods section).

Informed by Durlak and DuPre’s ecological framework for understanding effective implementation [1], and building on the results of Criss *et al.* [7], the aims of this study were to examine the community, provider, and organizational factors that explain the variation of media competition reach in school and afterschool programs, and describe the awareness of the media competition across other community sectors. To achieve this objective, the study team interviewed stakeholders directly involved with the media competition along with stakeholders who were engaged in the broader MA-CORD intervention to provide insight about community support of the media competition.

## 2. Methods

### 2.1. Theoretical Framework

**Figure 1:**
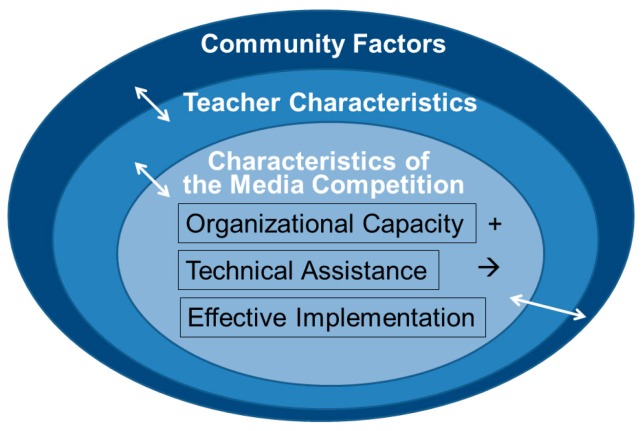
Ecological Framework for Understanding Effective Implementation of the Media Competition. Adapted from [1].

Since the media competition was nested within a multi-level intervention, we used Durlak and DuPre’s ecological framework [1] to guide the analysis of the media competition implementation. This framework asserts that effective implementation is influenced by variables in five categories: the prevention delivery system (features related to organizational capacity), the prevention support system (training and technical assistance), innovation characteristics (e.g., characteristics of the media competition), provider characteristics (e.g., teacher characteristics), and community factors. Figure 1 shows the ecological framework for understanding effective implementation of the media competition. All the factors can interact with each other, which creates a constellation of factor combinations that can lead to effective implementation. This framework provides clear guidance on focus areas to evaluate implementation.

### 2.2. Participants and Setting

There were two groups of stakeholders participating in two different types of key informant interviews. Media Competition Stakeholders consisted of teachers and staff from school and afterschool programs who had students participate in the media competition, and their interview focused solely on the media competition. MA-CORD stakeholders consisted of staff from schools, afterschool programs, clinics, WIC, the Park and Recreation Department, and community coalitions, and their interview focused on the broader MA-CORD intervention, including the media competition. MA-CORD stakeholders may or may not have had direct contact with the media competition and were included in order to understand views from various levels of participation.

All stakeholders were from Fitchburg and New Bedford. Both communities have a higher percentage of low-income residents and children classified as overweight or obese compared to the MA state-wide average [8]. In Fitchburg, six schools (6 out of 6 schools) and one afterschool program (1 out of 6 programs) had students submit entries to the media competition; and in New Bedford, nine schools (9 out of 23 schools) and two afterschool programs (2 out of 9 programs) had students submit entries. Criterion-based sampling [9] was employed to ensure the study team interviewed stakeholders from school and afterschool programs with various implementation levels, along with other stakeholders from other sectors. Teachers and staff submitted media competition entry forms on behalf of their students, and the study team contacted each teacher/staff who submitted a form to participate in an interview. The study team had access to a database with all MA-CORD stakeholder contact information. All stakeholders received at least two invitations to participate in an interview. Interviewees were compensated $40 for their participation. The Harvard T.H. Chan School of Public Health’s Institutional Review Board approved the protocol and interview guide for this study.

### 2.3. Instrument

The study team developed a standardized interview guide for both groups of stakeholders using the ecological framework for understanding effective implementation [1]. Table 1 lists the questions for media competition stakeholders, which focused on the media competition, and the corresponding questions for the MA-CORD stakeholders, which related to the broader MA-CORD intervention with questions specific to the media competition integrated into the guide. At the end of each interview, demographic information was captured including participant age range, race, ethnicity, and occupation. The interview guide was designed so that each interview would take approximately 30 min.

### 2.4. Data Collection and Analysis

All interviewers on the study team were trained in conducting qualitative interviews. The media competition stakeholders were interviewed in June 2013, immediately following the completion of the media competition and the announcement of competition results, and the MA-CORD stakeholders were interviewed between November 2013 and April 2014. One member of the research team directly contacted the prospective interviewees and conducted interviews with all media competition stakeholders. Two research team members interviewed the MA-CORD stakeholders. All interviews were conducted in English over the telephone except for two interviewees who submitted email responses. One telephone interview consisted of interviewing two stakeholders instead of one stakeholder. All telephone interviews were audiotaped and later transcribed.

Transcribed interviews were entered into NVivo 10 [10], a qualitative analytic software program. The study team coders were trained in qualitative data analysis and NVivo. The lead author developed the initial codebook using Durlak and DuPre’s framework. Additional codes were developed after reading all the interview transcripts for the media competition stakeholders. Two researchers coded two media competition transcripts together, then clarified operational definitions of codes and modified the codebook. Subsequently, the researchers independently coded the remaining media competition transcripts (five from each community) independently. The coders were able to compare the transcriptions to the original recordings. The initial inter-rater reliability was measured by a Kappa coefficient [11] in NVivo. The kappa scores for the major coding categories for the 10 transcripts were: funding (0.77), perceived need for media competition (0.94), perceived benefits of the competition (0.88), self-efficacy (0.93), compatibility (0.93), adaptability (0.60), staff buy-in (0.49), shared decision-making (0.64), communication (0.75), leadership/administrative support (0.80), and technical support (0.73). The researchers discussed discrepancies and re-coded the transcripts. The final inter-rater reliability among these transcripts ranged from a Kappa coefficient of 0.82 to 0.99 in all the major coding categories. A Kappa coefficient of >0.80 is considered excellent [12,13]. This benchmark facilitates a process for researchers to clarify their understanding of the data.

The second round of coding focused on the transcripts for the MA-CORD stakeholders. The lead author randomly selected five MA-CORD transcripts for independent coding using a random number generator. The two researchers coded the transcripts, which resulted with a Kappa coefficient of >0.80 in all major categories. The same codebook was used with fewer codes given the reduced number of questions in the MA-CORD interviews that focused on the media competition.

In total, over a quarter of the transcripts (28%, *n* = 15) were coded independently by two researchers (10 from the media competition and five from the MA-CORD stakeholder interviews). After researchers discussed discrepancies for all these transcripts, the lead author coded the remaining interviews and further discussed the analysis with the other researchers on the team.

For analysis, stakeholders were stratified by level of reach, which was defined as student participation rates within schools and afterschool programs (number of participants divided by number of all students in school/afterschool program). One level was no reach (no participation), which refers to school/afterschool programs that were eligible to participate and notified about the media competition but did not submit any entries. The other levels were low reach (<1%–3%), moderate reach （>3% with <100 participants; based on having a number of students that could connote at least the participation of one full class (e.g., at least 20 students in a school setting)), and high reach (>3% with ≥100 participants) [7]. The coders used a framework matrix in NVivo to analyze [14] differences across groups.

## 3. Results

### 3.1. Participant Characteristics

Table 2 has the demographic information for all stakeholders (*n* = 54) with 12 respondents participating in the media competition interview and the remaining 42 completing the integrated media competition questions with a larger MA-CORD intervention guide. Most stakeholders were white females with bachelor or master’s degrees who worked in the school sector as teachers or school nurses. Stakeholders were evenly dispersed between the two communities. Stakeholders from schools and afterschool programs (*n* = 38) represented organizations that demonstrated no (*n* = 6), low (*n* = 7), moderate (*n* = 16) and high (*n* = 5) reach for the media competition, along with school district employees (*n* = 4) who were categorized as “not applicable” reach because they were not part of a specific school. An additional 16 stakeholders represented non-school sectors.

### 3.2. Factors that Explain Variation in Implementation Effectiveness

The following section summarizes the framework’s five categories (organizational capacity, technical assistance, characteristics of the media competition, teacher characteristics, and community level factors) as factors affecting implementation of the media competition with examples provided for individuals from schools/afterschool programs (*n* = 38) with varying levels of program reach. In the following section, when “stakeholder” is preceded by low, moderate, or high reach; it connotes that stakeholder is from a school/afterschool program with that reach level (student participation level). In the quotations, FB represents Fitchburg, and NB represents New Bedford. The following themes were consistent among all reach levels, unless otherwise indicated. Common patterns will be shown, as well as, distinctions across reach levels. Results are shown to correspond with the media competition interview guide. However, it should be noted that high and moderate reach stakeholders reported the importance of staff commitment through having cascade of champions (Section 3.6). The authors coined the term “cascade of champions,” which adds to Rogers’ “champions” concept [15]. Cascade of champions refers to individuals who dedicate their personal influence to promote adoption of a program [15] on various organizational levels, such as starting with champions on the school district level to champions on the school administration level to champions on the teacher level. High and moderate reach stakeholders also reported the teacher’s adaptability to provide opportunity for students to participate in the media competition outside traditional class time (Section 3.5).

### 3.3. Community Level Factors

*Aware of resources*: Most stakeholders indicated that they were aware that the media competition was an initiative stemming from the school district office in partnership with MA-CORD program from the Massachusetts Department of Public Health and Harvard University. Stakeholders however reported mixed views on funding. Some low reach stakeholders indicated that funding was lacking; in contrast, high/moderate reach stakeholders indicated that they were aware of school district resources and/or had access to personal equipment/materials (e.g., iPhone): For example, one high reach stakeholder stated, “I had what I needed in terms of the artwork and that type of thing, but I knew that I could go to (MA-CORD School District Coordinator) if there was something I didn’t have so we were all set in that area” (elementary school teacher, 3rd–4th grade, high reach, FB).

*Parent/student excitement*: Several high/moderate reach stakeholders reported parent/student excitement: “they (parents) said that they knew their kids were working on it, and they were excited because the kids were excited about it” (elementary school teacher, 4th–5th grade, moderate reach, NB). In addition, high/moderate reach stakeholders reported that the parental support was critical in collecting media release forms and assisting their children with the competition at home.

### 3.4. Teacher Characteristics

*Relevant intervention*: The teachers in school and afterschool programs have varied views about the need for the media competition. Only one no reach stakeholder and one low reach stakeholder mentioned that the competition seemed relevant for their students. In contrast, high/moderate reach stakeholders indicated that the media competition was extremely relevant to addressing childhood obesity, and that it was an issue among their students and parents. One 4th grade teacher said, “It made the kids realize that they need to get more active. … They did not realize that they were in the house more than they were outside. … They just see what their parents do. So they just kind of follow along” (elementary school teacher, 4th grade, high reach, NB).

*Perceived benefits of the competition*: Most interviewees reported benefits of the media competition for their students, such as understanding the goals, critically thinking about their choices (e.g., hours of sleep, and food selection), behavior change, and excitement about the competition from the online presence and teaching their peers. High/moderate reach teachers who implemented the competition reported changed intentions and behaviors for their students concerning the MA-CORD goals. One middle school teacher said, “It (the media competition) really helped, and they started bringing water bottles and showing me. Then in the cafeteria, they are picking up their veggies and their fruits, and they would pass by me and show them to me. They were aware and they were actually doing the stuff” (middle school teacher, 5th–7th grade, moderate reach, FB).

Low stakeholders reported benefits from viewing the media competition as a source of health education materials *vs.* active participation in the media competition:

“I had quite a bit of the Portuguese population in my classroom last year. Portuguese people are big on soda with every meal. … Every parent got a booklet that Mass in Motion had sent to our school so every parent got a booklet. I tried talking about it in my newsletters and that kind of thing. I know some of the kids didn’t touch soda after that. Even if I got one not to drink soda again, I think it worked”.

(elementary school teacher, 5th grade, low reach, NB)

*Excitement from recognition*: Several high/moderate reach stakeholders mentioned that students were excited, and they felt a sense of school pride to see their work displayed at school, on the local community and television station. One elementary teacher described the students’ reaction to seeing their work on the Internet:

“It was like mania broke out in my classroom. … They shared with other students, it’s online! A couple of students saw schools that some of the people they know attend, and they were just excited. … I know my kids were coming back in school and saying that “my grandmother watched it in Portugal, and my uncle that lives here, he saw it and told me that I did a good job. They were really excited about that part”.

(elementary school teacher, 4th–5th grade, moderate reach, NB)

*Self-efficacy*: Across all reach levels, most stakeholders indicated that they felt equipped to facilitate the competition for their students because of previous engagement with the topic and training. One stakeholder said, “I think the wellness champion trainings were very helpful. … Once you have the information and the platform, the rest is easy. The kids’ level of engagement really determines the success of the event so I felt well prepared” (elementary school teacher, 2nd–4th grade, moderate reach, FB).

### 3.5. Characteristics of the Media Competition

*Compatibility*: Some high/moderate reach stakeholders indicated that the competition was a priority for the health and gym teachers. This elementary school teacher reported a similar view shared among other high/moderate stakeholders, “There are many changes going on with the district with healthier food offered in the cafeteria, as well as, summer programs that offer free meals. They’re healthy meals. There’s a big push to remove junk food at local vending with snack trucks and snacks shacks” (elementary school teacher, 2nd—4th grade, moderate reach, FB). Some low stakeholders indicated that the competition was not compatible with their current school plan (e.g., child wellness not addressed).

*Competing priorities*: All school and afterschool stakeholders indicated that the competition timeline was a barrier. Fitchburg had a three-month implementation timeline, and New Bedford requested an extension for a four-month implementation timeline. Many stakeholders reported that they would prefer to be given a longer time period so it would not conflict with statewide testing: “I would have liked to do it, but it’s just—it’s just a logistics problem. …The teachers are doing more than they can take right now trying to get the scores up, and the focus is testing, testing, testing and the academics.…Right now, the focus is we have to survive” (elementary school nurse, no entries, NB). Some no-entry and low reach stakeholders reported that they had additional required curricula (e.g., bullying) as well.

*Adaptability-Providing opportunity to participate in the media competition outside traditional class time*: This factor was very salient in engaging students to participate in the media competition. Several high/moderate reach stakeholders were able to dedicate unconventional time to the competition, and the flexibility in the implementation of the media competition opened various ways to allow students to participate. One high elementary teaching team in New Bedford created an afterschool program for two weeks so that their students could participate in the competition. A middle school principal reported, “It (participating in the media competition) really just came down to access. Part of this was done after school. Part of it was done during the school day. I think there was a component, actually, where children were coming in on the weekends. That was—pulling all of that together, the kids had access to it” (middle school principal, 5th–6th and 8th grade, moderate reach, FB).

### 3.6. Organizational Capacity

*Cascade of champions*: A cascade of champions refers to having program champions on various organizational levels (e.g., starting from the school district coordinator to school principals to teachers). It seemed to be the most salient facilitator in successfully implementing the media competition. High/moderate reach stakeholders reported the layers of support from committed staff—the MA-CORD school district coordinator (SDC), school principals, and teachers. Most stakeholders from both communities reported that the SDC was “awesome”, visited each school to introduce the media competition, helped staff submit entries, and communicated with them throughout the entire process (sometimes sending 2–3 emails a day).

The school administration was involved on varying levels. Across reach levels, several stakeholders mentioned they had to get approval from the school principal, especially since some students would appear on the Internet with SchoolTube, similar to YouTube with the addition of teacher moderation. Several moderate reach stakeholders indicated that their principal was supportive: “My principal is very good at programs like this so he was aware of it and he was ready to supply everything that I needed” (middle school teacher, 5th–7th grade, moderate reach, FB). A few moderate reach stakeholders indicated that they would have preferred more support from their principal.

Most high/moderate reach stakeholders reported that teacher champions for the media competition were critical for activating student engagement. Regardless of staff buy-in, teacher champions were dedicated to assisting their children with the media competition. One stakeholder said, “I made time in my own class for kids to work on it, but I don’t feel as a whole staff that many teachers really valued it or supported to the level” (middle school teacher, 5th–7th grade, moderate reach, FB).

Several moderate reach stakeholders indicated that they received support from the specialists at their school, such as the art, music, gym, or health teacher, school nurse, or wellness champion. The teacher champions provided opportunities outside of traditional class time for students to contribute to media competition (e.g., created an afterschool program). In addition, one high reach school had the entire 4th grade team commit to conducting the media competition. Those teachers supported each other and garnered support from the school administration and other teachers in the voting process: “(After the school district coordinator provided guidance), the entire staff (including administration) definitely helped us out (4th grade teacher team). They all came to the forum to watch the videos, to vote, and to cheer us on” (elementary school teacher, 4th grade, high reach, NB).

No/low reach stakeholders reported that staff members did not take the lead on facilitating the media competition due to a lack of interest in the competition or needing more outside organizational support. A stakeholder said, “I just know that some of the teachers were not interested, and they didn’t do anything with it” (elementary school teacher, No entries, NB).

### 3.7. Technical Assistance

Across stakeholders, there was no pattern of variation in technical assistance needs by reach. About half of the stakeholders reported that they did not need any technical assistance. The other half needed assistance with SchoolTube and shared that the School District Coordinator, media competition manager, and SchoolTube support staff were helpful with the process. A few stakeholders reported that they would have liked more support in engaging student participation during the onset of the competition. Stakeholders in Fitchburg shared how the guidelines changed from the community meeting stage to implementation, and they would have preferred a clearer explanation of the changes and listing of the prizes (the prizes were not listed based upon school district policy).

### 3.8. Awareness of the Media Competition across Community Sectors

Eleven out of 42 MA-CORD stakeholders had not heard of the media competition (no stakeholders from the WIC and Parks and Recreation Department sector; two from the clinic sector; and three from the school sector (one was new to his/her position at a school with no entries)). MA-CORD stakeholders from the clinic and coalitions (*n* = 16) in particular had a very positive perception of the media competition. A coalition member stated, “I think the media competition was a success (especially since we integrated) it into the website (to create) community-wide voting. I thought that was really successful because we’ve had over 500 people vote through our website, which I thought was fantastic” (Coalition, NB).

Several stakeholders from non-school/afterschool sectors reported that they thought the media competition could be helpful to address childhood obesity. A clinician staff reported, “…the biggest driver is that these kids are now being made aware that it’s okay if they need a stretch break, they can get up and stretch, and when they come back, they’re expected to come back and be ready to then learn. I think it’s definitely catching on. I think the teachers are using it in a positive way. I think it’s only going to get better” (Clinic Staff, N/A Participation, FB). Some stakeholders indicated that they knew about the competition from their own children attending a school or afterschool program that was participating.

## 4. Discussion

Durlak and DuPre’s framework components were applied to ascertain the key factors for effective implementation between differing levels of program reach in school and afterschool programs. In this study, higher reach indicates success because more students had the opportunity to learn about the target behaviors that reduce childhood obesity. Organizational capacity of committed teachers/staff and adaptability of the media competition seemed to be drivers for higher reach within school and afterschool programs; specifically, having a cascade of champions and providing opportunity to participate in the media competition outside traditional class time.

The implementation of a student media competition with video, lyrics, and artwork puts high demands on teachers who are grappling with competing academic demands and priorities. Therefore, the need for a champion was paramount. At the onset of the study, MA-CORD established Wellness Champions at each school as part of the broader MA-CORD intervention to teach the evidence-based lessons and implement school wellness policies. However, not all Wellness Champions translated into Media Competition Champions. When teachers feel that their work is meaningful and beneficial to their students, then they feel motivated to implement the intervention [16]. Therefore, it is important to identify champions who are passionate about the specific innovation because their passion in turn encourages adoption of an innovation by others [15]. For this particular competition, it would have been helpful to target more human resources toward recruiting teachers who expressed a specific interest in the creative process and subject matter.

Program champions have been shown to ease the implementation of health promotion programs including programs focusing on childhood obesity prevention in school settings [17,18] along with community marketing [19]. While champions are typically middle-level staff [20], our study highlights the importance of having a cascade of champions, or champions across organizational levels. Schools with moderate participation consistently spoke about the school district coordinator’s enthusiasm and active engagement, their school administration’s backing and involvement, and their own fortitude to encourage their students to develop projects and even provide their personal resources to contribute to the students’ success. Since program champions can influence the use of evidence-based prevention programs in schools [21,22,23,24], this structure of a cascade of champions could offer a new conceptualization of how to frame implementation in a multi-level intervention. Program planners could specifically set-up a cascade of champions in the intervention design so that there is a multi-level team approach to provide support bi-directionally throughout various ecological levels of the organization (e.g., teacher, administration) and its partner organizations (e.g., school district office).

The media competition was designed such that schools and afterschool programs had the autonomy to implement the competition to fit within their program schedule. We observed that teachers who provided students with opportunities to participate in the media competition outside traditional class time, had higher levels of participation. In addition, teachers could choose to have students submit in all categories (*i.e.*, video, lyrics, and artwork) or just one. The community context also affected implementation of the media competition. For instance, Fitchburg had a small school district so the school district coordinator was able to actively communicate with all schools, but the school district policy concerning finances restricted the number of student winners. New Bedford had a larger school district and was in a state of fluctuation during the competition due to a transition of superintendents and staffing concerns. The school district coordinator requested that the timeline be shifted so that more teachers could be rallied to participate. Adaptability gave the teachers the flexibility to modify the media competition to fit their preference [1], but adaptability is missing from many frameworks and checklists that assess intervention applicability and transferability [25].

### Limitations

We did not test the effectiveness of the media competition independent of the other intervention activities of MA-CORD, yet we were able to collect implementation data to inform future iterations of the media competition. The media competition interviews consisted mostly of teachers with moderate reach so they could have been more positive about the program implementation compared to the stakeholders representing no entries or low participation who were not represented. It was helpful that some of the under-represented participants were able to contribute in the MA-CORD stakeholder interviews. Since the media competition only had three afterschool programs submit to the competition with one person participating in the detailed media competition interview, the afterschool perspective did not reach saturation. However, the school sector did reach saturation [26]. The interviewers were all part of the MA-CORD study team so there was a potential for social desirability bias in that the respondents may have provided information that they perceived the interviewers wanted to hear. To counter this bias, the interviewers shared that the respondent’s comments would be used for program improvement and that identifying information would be removed. In addition, many stakeholders were involved in the planning process of MA-CORD sector interventions so it is feasible that they saw the value of their previous feedback. In addition, the study findings apply directly to the two communities where the intervention was conducted. The results may not be generalizable in other types of communities with different populations, but the findings could be applicable to similar communities.

## 5. Conclusions

Utilizing Durlak and DuPre’s ecological framework for understanding effective implementation allows this study to contribute to the field of implementation science by identifying key factors to support intervention participation. Specifically, our findings introduced the concept of cascade of champions, which can offer a new perspective on intervention design and a recommended direction for further study. Since the media competition was part of a multi-level, multi-sector intervention, these results will add to the ability to triangulate data concerning the effectiveness of MA-CORD, as well as, evidence to facilitate program implementation in other multi-level community interventions.

## Figures and Tables

**Table 1:** Media Competition Interview Guide.

**Interview Guide for the Media Competition Interviews**
(Participants = teachers/staff who submitted a media competition entry)
**Construct #1: Community Level Factors**
(1) Tell me about how Mass in Motion Kids (MiM Kids) * School Media Competition is perceived in your community.
(2) Describe the support that you received in your school regarding the media competition. (Prompt: Did you have enough time?)
(3) What about funding? Please explain.
**Construct #2: Teacher Characteristics**
(1) How was the media competition relevant to addressing childhood obesity in your (school/afterschool program)?
(2) What type of benefits do you think the media competition will achieve within the (school/afterschool) level?
(3) How well equipped did you feel to help students participate in the competition?
**Construct #3: Characteristics of the Media Competition**
(1) How well did the media competition fit your school’s (school’s/afterschool’s) mission and current priorities? Please explain.
(2) How could the media competition have been adapted to fit into your (school/afterschool) norms?
**Construct #4: Organizational Capacity**
(1) What factors at your (school/afterschool program) contributed to implementing the media competition? (Prompts: Organizational factors: work climate, organizational norms regarding change, integration of new programming, staff buy-in)
(2) Who decided how the media competition would be implemented in your (school/afterschool program)? Were other people in the (school/afterschool program) supportive of the decision-making? Please explain.
(3) What type of support did you receive from the administration?
**Construct #5: Technical Assistance**
(1) Once the competition was underway, what technical support did you receive from *MiM Kids* at your school? Was this sufficient to meet your needs? What resources would have been helpful?
**Interview Guide for the MA-CORD Stakeholders ^+^**
(Participants = individuals from all sectors of the MA-CORD intervention, including those who did not have direct experience with the media competition)
(1) Tell me about how *MiM Kids* School Media Competition was viewed in your community.
(2) What type of benefits do you think the media competition had for the schools and afterschool programs? What about benefits for the students?
(3) How well equipped did you feel to help schools participate in the competition? Explain.
(4) What are your thoughts about the implementation process for the media competition?
(5) How could have the implementation been improved?

* Massachusetts Childhood Obesity Research Demonstration (MA-CORD) was known as *Mass In Motion Kids (MiM Kids)* in the community; + These questions are a sub-set from media competition interview guide because they were incorporated into an interview guide about all aspects of the MA-CORD intervention.

**Table 2:** Media Competition Interview Participation Demographic Information.

Variables (% (n))	Total *n* = 54		Media Competition Stakeholders *n* = 12		MA-CORD Stakeholders *n* = 42	
Sex						
Female	93%	(50)	100%	12	90%	38
Male	7%	(4)	-	0	10%	4
Age Category						
18–29	7%	(4)	17%	2	5%	2
30–39	20%	(11)	33%	4	17%	7
40–49	24%	(13)	33%	4	21%	9
50–59	35%	(19)	17%	2	40%	17
60 or older	11%	(6)	-	0	14%	6
Not specified	2%	(1)	-	0	2%	1
Race						
White	87%	(47)	92%	11	86%	36
Black/African American	6%	(3)	8%	1	5%	2
Asian	2%	(1)	-	0	2%	1
Other-Hispanic	4%	(2)	-	0	5%	2
Not specified	2%	(1)	-	0	2%	1
Highest degree earned						
High School	2%	(1)	-	0	2%	1
Associate’s Degree	6%	(3)	8%	1	5%	2
Bachelor	30%	(16)	17%	2	33%	14
Master	56%	(30)	75%	9	50%	21
Doctoral, MD	6%	(3)	-	0	7%	3
Not specified	2%	(1)	-	0	2%	1
Sector						
School	59%	(32)	92%	11	50%	21
Afterschool	11%	(6)	8%	1	12%	5
Clinic	15%	(8)	-	0	19%	8
WIC	6%	(3)	-	0	7%	3
Parks and Recreation	4%	(2)	-	0	5%	2
Coalition	6%	(3)	-	0	7%	3
Job Title						
School teacher	30%	(16)	100%	12	10%	4
School nurse	20%	(11)	-	0	26%	11
School administrators	9%	(5)	-	0	12%	5
Clinic staff	15%	(8)	-	0	19%	8
Afterschool staff	11%	(6)	-	0	14%	6
Parks and Recreation Staff	4%	(2)	-	0	5%	2
Coalition Members and School District Coordinators	6%	(3)	-	0	7%	3
WIC staff	6%	(3)	-	0	7%	3
Community						
Fitchburg	46%	(25)	50%	6	45%	19
New Bedford	54%	(29)	50%	6	55%	23
Media Competition Reach (Participation Level) ^a^						
No entries	11%	(6)	-	0	14%	6
Low	13%	(7)	8%	1	14%	6
Moderate	30%	(16)	50%	6	24%	10
High	9%	(5)	42%	5	0	0
Not applicable *	37%	(20)	-	0	48%	20

^a^ Participation Level = Low = <1%–3% of student participation in school (School Range: 1–11 students; Afterschool Range: 3 students); Moderate = >3% with <100 participants (School Range: 23–50 students; Afterschool Range: 5–15 students); High = >3% with >100 participants (School Range: 120–192). * Not applicable = 4 school district employees not attached to a specific school; 16 non-school/afterschool sector stakeholders. Note: Percentages may not add to 100% based on rounding.
